# Hepatocyte ploidy in cats with and without hepatocellular carcinoma

**DOI:** 10.1186/s12917-021-02812-1

**Published:** 2021-03-04

**Authors:** Jacqueline Post, Ingeborg M. Langohr, Cynthia R.L. Webster, Peter Mottram, Chin-Chi Liu, Andrea Johnston

**Affiliations:** 1 Veterinary Clinical Sciences, School of Veterinary Medicine, Louisiana State University, LA Baton Rouge, USA; 2 Pathobiological Sciences, School of Veterinary Medicine, Louisiana State University, LA Baton Rouge, USA; 3 Veterinary Clinical Sciences, Cummings School of Veterinary Medicine, Tufts University, MA North Grafton, USA

**Keywords:** Ploidy, Liver, Hepatocellular carcinoma, Feline

## Abstract

**Background:**

Domestic cats rarely develop hepatocellular carcinoma. The reason for the low prevalence is unknown. Reductions in hepatocellular ploidy have been associated with hepatic carcinogenesis. Recent work in mice has shown that livers with more polyploid hepatocytes are protected against the development of hepatocellular carcinoma. Hepatocyte ploidy in the domestic cat has not been evaluated. We hypothesized that ploidy would be reduced in peri-tumoral and neoplastic hepatocytes compared to normal feline hepatocytes. Using integrated fluorescence microscopy, we quantified the spectra of ploidy in hepatocellular carcinoma and healthy control tissue from paraffin embedded tissue sections.

**Results:**

Feline hepatocytes are predominantly mononuclear and the number of nuclei per hepatocyte did not differ significantly between groups. Normal cats have a greater number of tetraploid hepatocytes than cats with hepatocellular carcinoma.

**Conclusions:**

Total hepatocellular polyploidy in normal cat liver is consistent with values reported in humans, yet cellular ploidy (nuclei per cell) is greater in humans than in cats. Tetraploid cat hepatocytes are predominantly mononuclear.

## Background

Most mammalian cells are diploid (2n), but some cells including cardiac myocytes, megakaryocytes, and hepatocytes can contain more than two homologous chromosomes. Polyploidy is defined by nuclear and cellular DNA content. Nuclear polyploidy refers to an increase in the number of chromosomes per nucleus and cellular polyploidy is an increase in the number of nuclei per cell [1, 2]. The degree of polyploidization varies among mammals; in murine species 75–90 % of hepatocytes are polyploid whereas in adult humans the number of polyploid cells averages 20–45 % [3–5]. Polyploidization or whole genome amplification arises due to failed cytokinesis or, less often, endoreplication [2, 6–9]. The polyploid state of the liver is changeable, particularly during development and instances of cellular stress. [4, 10]. Polyploidy is essential to reparative regeneration in many organs, but has also been associated with genome instability and tumorigenesis when polyploid hepatocytes undergo mitosis [11–13]. Yet, recent work in mice has shown that hepatocellular polyploidy (> 2n) suppresses tumor development [4, 14]. Polyploidy may serve a hepatoprotective purpose by limiting oxidative stress, genotoxic damage, or by limiting tumor-suppressor loss of heterozygosity [3, 4, 10].

In humans, hepatocellular carcinoma (HCC) is the most common type of liver cancer and a leading cause of cancer related-death [15–17]. The risk factors for HCC in humans include cirrhosis, viral hepatitis, non-alcoholic fatty liver disease, and hepatotoxicosis. The role of ploidy in the development of human hepatocellular carcinogenesis is less clear than in mice and may be largely dependent on the context. Although a reduction of ploidy has been identified in human pre-neoplastic liver nodules, tetraploidy can lead to chromosomal instability and aneuploidy [3, 18]. Unlike humans, primary hepatic tumors in the domestic cat are rare with an estimated prevalence ranging from 1 to 3 % of all feline cancers [19–21]. Survival statistics for cats predict a median survival of 1.4 years following diagnosis. Life expectancy improves to 2.4 years when surgical excision is possible [20]. The etiology of feline HCC is ill-defined and has not been definitively linked to viral disease or hepatic lipidosis [20, 21]. We hypothesized that the species differences in ploidy could account for the lower frequency of hepatocellular cancer in cats. The polyploid state of feline liver has not been reported. Our objective was to quantify hepatocellular ploidy in a cohort of cats with hepatocellular carcinoma and matched normal control cats.

## Results

**Fig. 1:**
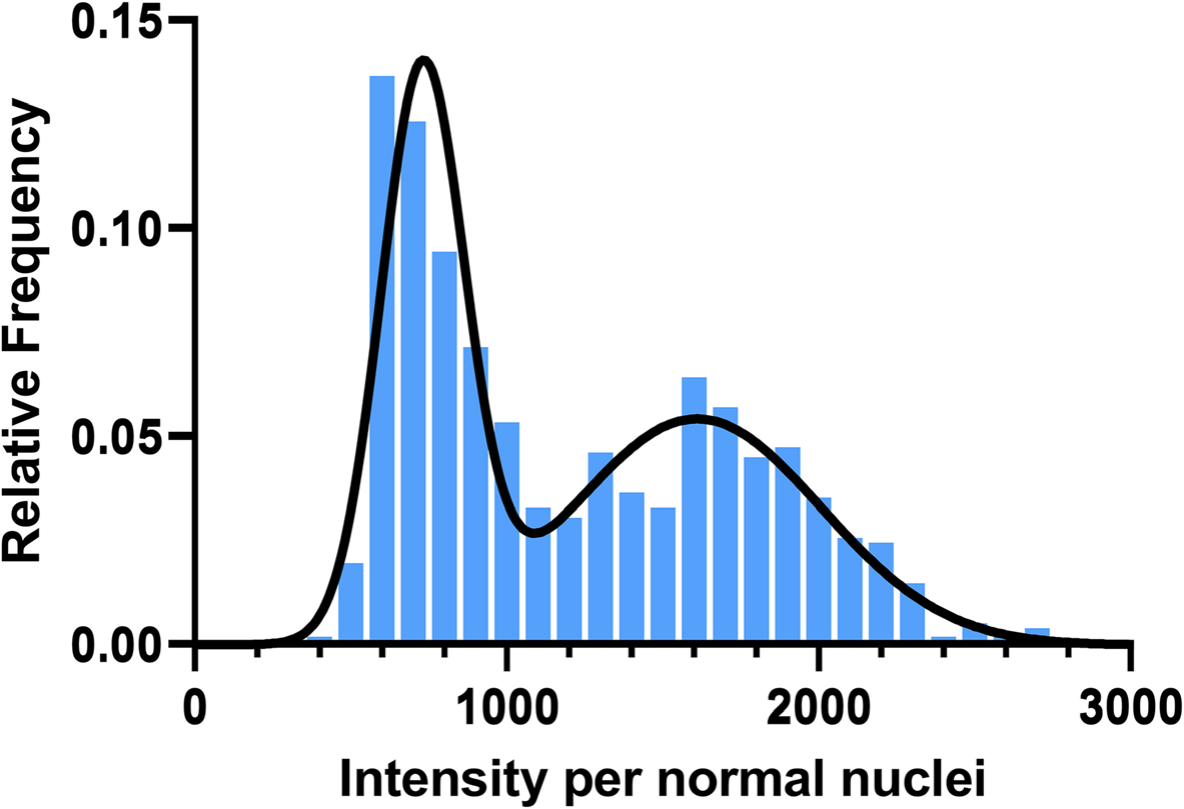
Histogram of relative DNA intensity distribution curve per normal nuclei via Gaussian curve. Gaussian mixture distribution with 2 components in 1 dimension. Fluorescence intensity for normal hepatocyte nuclei (x-axis) is compared to the relative frequency (y-axis) to determine nuclear ploidy. The black line outlines the fit in Gaussian mixture distribution using “fitgmist” package from MATLAB *ver R2020a*, with the first peak (less than 1095 ms) corresponding to 2n hepatocytes and the second peak (greater than 1095 ms) corresponding to 4n hepatocytes

**Fig. 2:**
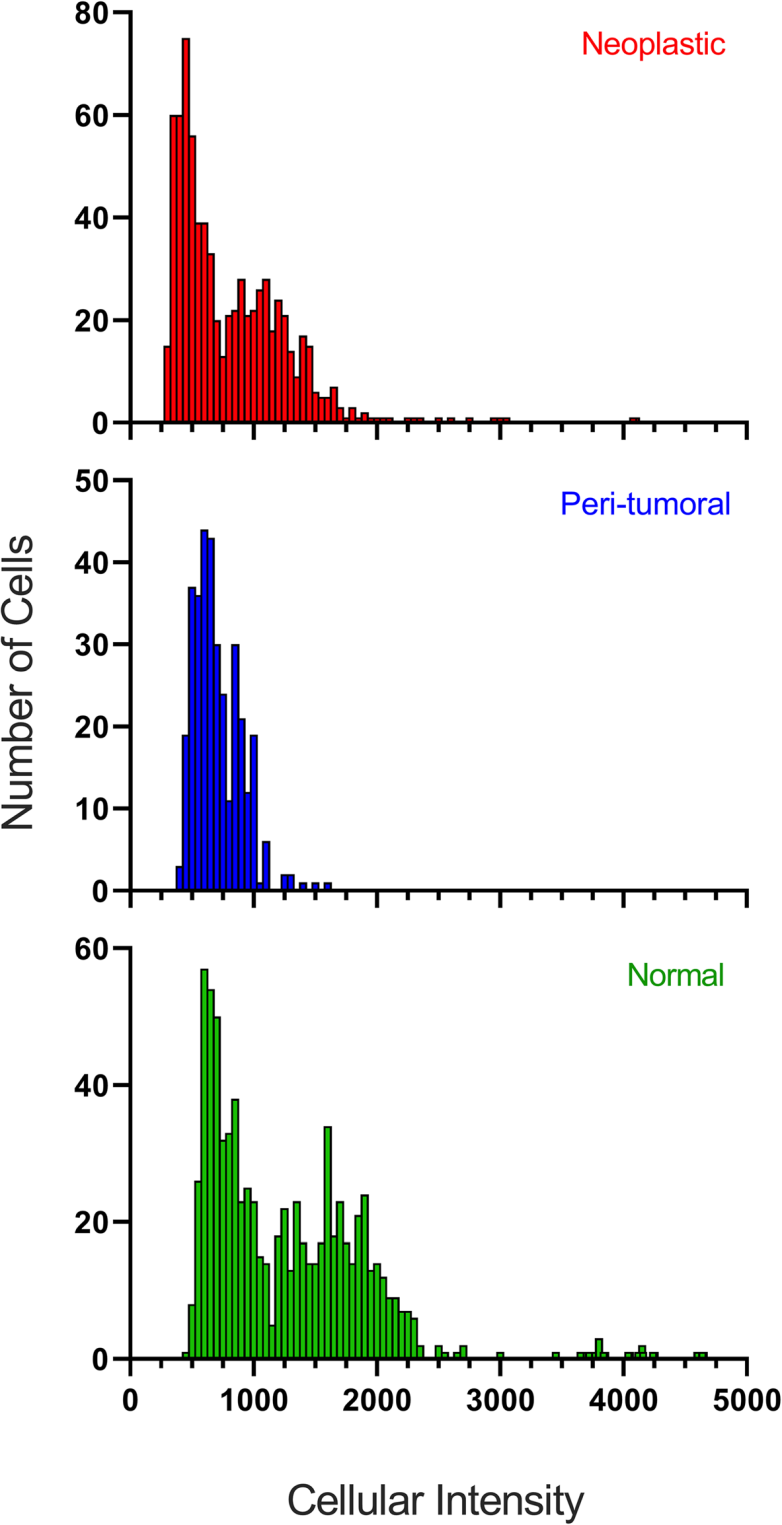
Histogram intensities for neoplastic, peri-tumoral, and normal hepatocytes. The frequency distribution for individual nuclear H42 staining intensities per each hepatocyte (bin: 50). The top graph represents neoplastic hepatocytes, the middle peri-tumoral hepatocytes, and the bottom normal hepatocytes. The bars represent the number of cells displaying an intensity in the given ranges (y-axis). The higher the bar, the more cells showed that intensity reading. The intensity distributions within each cell, among three different cell types, were compared using Kolmogorov-Smirnov test and normal feline liver polyploidy was significantly different than peri-tumoral (P < 0.0001) and neoplastic liver ploidy (*p* < 0.0001). The peaks of the normal hepatocytes (top) match those from Fig. 1; however, Fig. 1 shows intensity values per nuclei of the normal hepatocytes and Fig. 2 displays intensity values per cell for all hepatocyte types

**Fig. 3:**
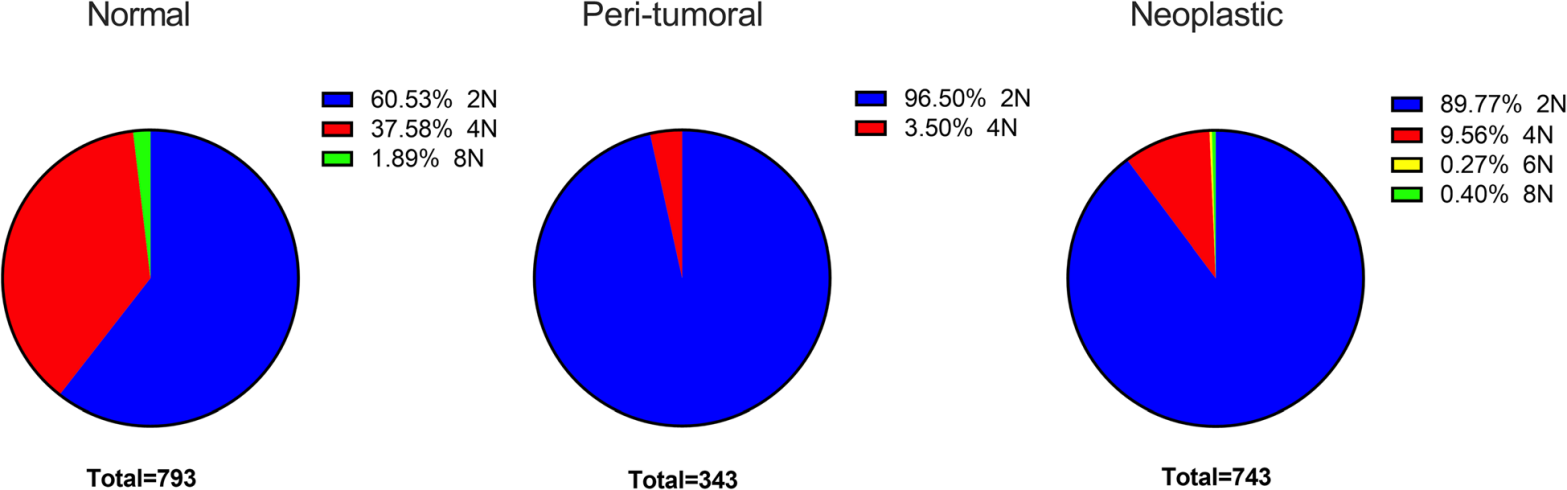
Ploidy count per hepatocyte type. Pie graphs display ploidy per cell for normal, peri-tumoral, and neoplastic feline liver. Percentage of diploid hepatocytes and polyploid hepatocytes are indicated by color. The 6n population in neoplastic tissue may represent an aneuploid population or inaccurate distribution cutoff between 4n and 8n due to low cell numbers in the > 4n subset. Normal hepatocytes (*n* = 793 cells), peri-tumoral (*n* = 343 cells), and neoplastic (*n* = 743 cells). The ploidy distributions within each cell, among three different cell types, were compared using Kolmogorov-Smirnov test and normal feline liver polyploidy was significantly greater than peri-tumoral (*P* < 0.0001) and neoplastic liver ploidy (*p* < 0.0001)

**Fig. 4:**
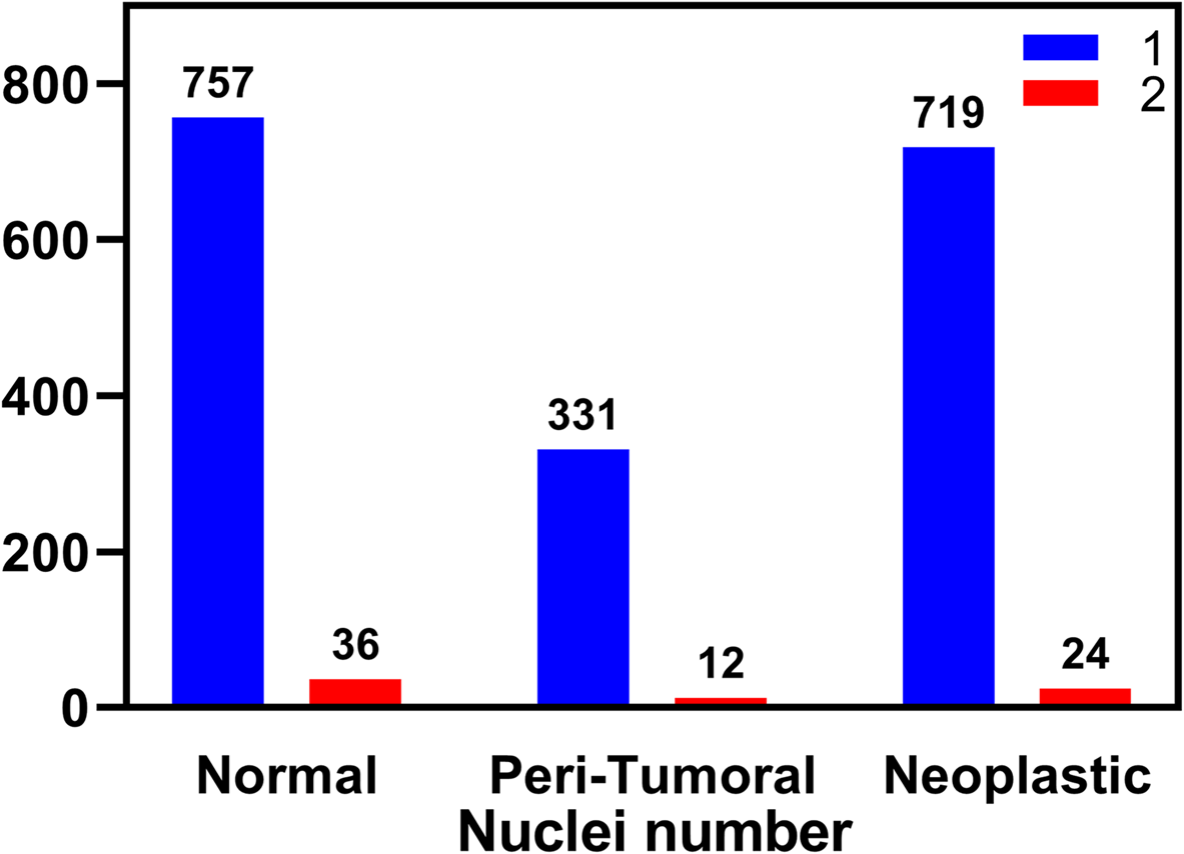
Nuclei number per hepatocyte. Mono- or binucleated cells were quantified for normal hepatocytes, peri-tumoral hepatocytes, and neoplastic hepatocytes (x-axis). The y-axis represents the number of cells with one or more than one nucleus. The number of nuclei per hepatocyte did not differ significantly between groups (*p* > 0.05, chi-squared test)

A Gaussian mixture curve was used to show the relative frequency of intensity distribution for all normal nuclei measured (Fig. 1). The peaks, 730 ms and 1610 ms, correspond to the average H42 intensity reading for 2n and 4n. The overlap of the two curves was determined to be 1095 ms. Intensities less than 1095 ms were considered 2n and values over 1095 ms were 4n. Raw intensities per cell are shown in Fig. 2. Normal feline liver polyploidy was significantly greater than peri-tumoral (P < 0.0001) and neoplastic liver ploidy (p < 0.0001; Fig. 3). In the normal cohort, the percentage of polyploid hepatocytes in normal feline liver was 39.47 %. Only 10.23 % of neoplastic hepatocytes were polyploid. Feline hepatocellular polyploidy is similar to values reported in humans (30–50 %), but less than mice (75–90 %) [1, 3, 5, 22]. The number of nuclei per hepatocyte did not differ significantly between groups (p > 0.05; normal − 4.5 %, peri-tumoral – 3.5 %, or neoplastic − 3.2 %, Fig. 4). Mononuclear tetraploid hepatocytes compose 30.6 % of normal feline hepatoctyes, 28.5 % of peri-tumoral hepatocytes, and only 7.4 % of neoplastic hepatocytes.

## Discussion

In the present study, feline hepatocellular ploidy was quantified in hepatocellular carcinoma, peri-tumoral hepatocytes, and hepatocytes of age- and gender-matched, normal controls. In the normal cohort, feline hepatocytes had a greater number of polyploid hepatocytes than tumoral or peri-tumoral hepatocytes. Due to the observational nature of this study, we cannot determine whether decreased liver ploidy plays a causal role in tumorigenesis; yet, this hypothesis is supported by research in mice. Genomic analysis has demonstrated that mutations in key oncogenes and tumor suppressors play a role in the pathogenesis of HCC [15]. In mice, 90 % of hepatocytes are hyper-diploid and genetic depletion of hepatocellular ploidy increases the development of HCC [22]. Loss of one tumor suppressor copy in a diploid cell leads to loss of heterozygosity, promoting potential for neoplastic transformation [22, 23]. Work by Kreutx et al.. identified unique metabolic characteristics dependent on nuclear ploidy in mice [5]. Differential gene expression and decreased insulin binding were found in polyploid nuclei compared to diploid nuclei. These differences may also contribute to altered mutagenesis.

Mononuclear tetraploid hepatocytes were the major component of the polyploid fraction in normal feline liver. Although total feline hepatocellular ploidy is similar to humans, the chromosomal distribution between nuclei differs [1, 3, 18, 24–26]. Normal human liver has a greater number of binuclear tetraploid hepatocytes but mononuclear tetraploidy is amplified in HCC [1]. In feline peri-tumoral and tumoral tissue, the majority of hepatocytes are mononuclear diploid. Whether this difference influences differences in feline HCC tumorigenesis remains to be determined. The mononuclear tetraploid phenotype suggest that these cells generated from either endoreplication or mitotic slippage, rather than cytokinesis failure [2]. Endoreplication produces terminally differentiated cells that are non-proliferating but potentially predisposed to tumorigenic transformation.[11, 27, 28].

The rarity of HCC in the domestic cats limited sample numbers and the power of this study. Despite searching archives at two universities, only 7 cases of feline HCC were identified. Despite its small-scale, this research identified unique features of feline hepatocellular ploidy that may be broadly applicable to hepatocellular carcinogenesis. In humans, several other risk factors contribute to the development of HCC, including chronic viral hepatitis, exposure to toxins, and fatty liver disease [15]. The etiology of feline HCC is unclear [20, 21]. Recent studies have correlated feline HCC to hepadnavirus infection, which bears further investigation [29]. Hepatitis B virus, a well-defined cause of human HCC, is a member of the hepadnavirus family and has been reported to alter hepatocyte ploidy [10, 30]. Future studies will assess risk factors known to contribute to the development of HCC in cats and evaluate hepatocyte ploidy in a larger number of normal cats [15].

## Conclusions

Feline hepatocytes are predominantly mononuclear and cellular ploidy does not differ significantly between healthy, peri-tumoral, or neoplastic liver. Normal cat liver has a significantly greater number of 4n hepatocytes than cats with HCC. Total hepatocellular polyploidy in normal cat liver is consistent with values reported in humans, yet cellular ploidy (nuclei per cell) is greater in humans than in cats. Tetraploid cat hepatocytes are predominantly mononuclear. Neoplastic feline liver has a greater number of mononuclear, diploid hepatocytes than normal liver. This may be relevant in regards to the hepatocellular tumorigenesis in cats.

## Methods

### Sample selection

 Feline hepatocellular carcinoma specimens were selected from archival histology libraries at Tufts University, Cummings School of Veterinary Medicine and Louisiana State University (LSU) School of Veterinary Medicine, Louisiana Animal Disease Diagnostic Laboratory (LADDL). Seven cases were identified (Table 1). Additionally, 7 sex (3 male, 4 female) and age-matched (8 to 14 years), control cases with normal hepatic histology were selected from the LADDL archives. The formalin-fixed, paraffin-embedded liver specimens were sectioned and stained with hematoxylin & eosin (H&E). Slides were reviewed for diagnostic criteria by a veterinary anatomic pathologist (IML). Samples that were morphologically classified as hepatocellular carcinoma were scored according to the World Health Organization and the Edmonson and Steiner grading classifications (Table 1) [31, 32]. Matched controls samples were deemed free of steatosis and inflammatory cells, which could influence hepatocellular ploidy.

### Immunofluorescence staining protocol

Five µm sections were cut and mounted on charged slides. Samples were deparaffinized in xylene and serially rehydrated using a descending gradient of ethanol-water solutions. Slides were washed in phosphate buffered saline with 0.1 % Triton X-100 (PBST). After citrate buffered antigen retrieval, tissues were blocked with 5 % normal goat serum at room temperature for one hour. Tissues were incubated with β-catenin primary antibody (1:200, Invitrogen Beta-catenin polyclonal antibody; Carlsbad, CA) in 1 % in normal goat serum at room temperature for 2 hours, washed with PBST, and incubated with secondary antibody (Biotium CF594 F(ab’) 1:1000; Fremont, CA) for 1 hour at room temperature. Hoechst 33,342 (H42) nuclear stain (1ug/mL, Thermo Scientific) was applied for 20 minutes at room temperature. After dye incubation, slides were washed for 5 minutes with PBS. Slides were cover slipped using the Biotium EverBrite Hardset Mounting Medium (Fremont, CA) and allowed to dry for a minimum of 30 minutes before microscopic analysis.

**Table 1:** Signalment of cats with hepatocellular carcinoma and tumor grade

Gender	Age (years)	WHO Grade	Edmondson & Steiner Grade
Male neutered	8	Moderately differentiated	Grade II
Male neutered	11	Moderately differentiated	Grade II
Male neutered	14	Well differentiated	Grade I
Female spayed	8	Moderately differentiated	Grade II
Female spayed	12	Well differentiated	Grade I
Female spayed	13	Poorly differentiated	Grade III
Female spayed	18	Moderately differentiated	Grade II

### Microscopic Analysis

**Fig. 5:**
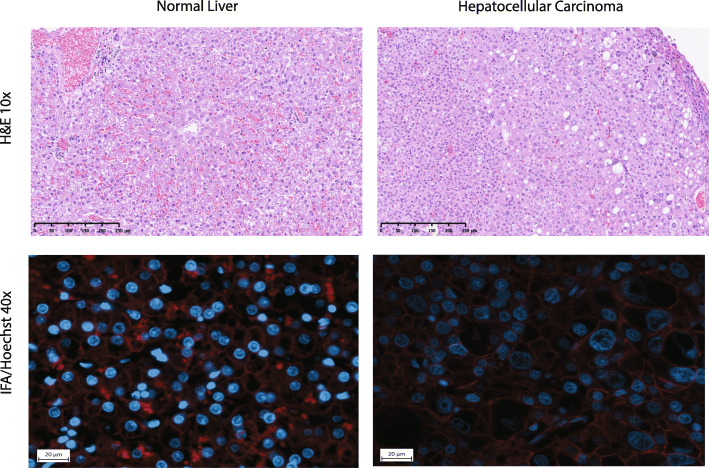
Representative images of histology and immunofluorescent staining of normal liver and hepatocellular carcinoma. (Top) Hematoxylin & eosin stain of normal feline liver (left) and feline HCC (right). (Bottom) Immunofluorescent antibody (IFA) images of normal feline liver (left) and feline HCC (right) stained with Hoechst nuclear stain (blue) and Beta-catenin IFA (red)

Modeled after Toyoda et al. 2005, slides were examined using integrated fluorescence microscopy (Zeiss AXIO Observer Z1, Carl Zeiss, Göttingen, Germany). The Zeiss Neofluar 40X/0.75 EC plan objective (Fluorescence, high transmission) was used for all images acquired. The Zeiss #64 filter set was used to image β-catenin (Excitation:587/25 and Emission: 647/70). Hoechst nuclear imaging was performed using Zeiss #34 filter set (Excitation 390/22 and Emission 460/50). To ensure that the same cells were not counted twice, slides were read in a systematic manner, moving from top right to left of the slide and then on successive descending lines. Each captured image contained approximately 25 square mm of surface area. Digital images were collected and merged with Zen2 software (Zen Pro, Fig. 5).

### Ploidy measurement

 Quantification of cellular ploidy (mono- or binucleate) was enabled by β-catenin immunofluorescent staining to outline the plasma membrane. Nuclear ploidy (chromosome number per nucleus) was quantified using H42 staining. H42 stoichiometrically binds to the minor groove of DNA when crosslinking fixatives are used. A minimum of 100 cells per section were analyzed. Cells were excluded if they displayed overlapping nuclei or indeterminate plasma membrane borders.

### Statistical analysis

 A diploid feline cell contains 38 chromosomes (19 pairs), differing from mice and humans, thus a species specific intensity distribution was generated. The individual nuclear intensities were combined in multinucleated cells to represent the total cellular ploidy. Measurements from histologically normal feline liver were compiled and graphed based on intensity per nuclei and probability density. The cutoff intensity for cell ploidy was determined by fitting two Gaussian mixture with “fitgmist” package from MATLAB *ver R2020a*. All other analyses were performed with JMP Pro 15 (SAS Institute Inc., Cary, NC). The intensity and ploidy distributions within each cell, among three different cell types, were compared using Kolmogorov-Smirnov test (Biesterfeld et al. 1994). Nuclei number and cell type was compared via chi-squared test. *P* < 0.05 was considered significant.

## Data Availability

The datasets used during the current study are available from the corresponding author on reasonable request.

## Abbreviations

H42: Hoechst 33342.
HCC: Hepatocellular carcinoma.
ms: Milliseconds.
PBST: Phosphate Buffered Saline + 0.1 % Triton X-100.

## References

[1] Bou-Nader M, Caruso S, Donne R, Celton-Morizur S, Calderaro J, Gentric G (2020). Polyploidy spectrum: a new marker in HCC classification. Gut.

[2] Gentric G, Desdouets C (2014). Polyploidization in liver tissue. Am J Pathol.

[3] Wang MJ, Chen F, Lau JTY, Hu YP (2017). Hepatocyte polyploidization and its association with pathophysiological processes. Cell Death Dis.

[4] Zhang S, Zhou K, Luo X, Li L, Tu HC, Sehgal A (2018). The Polyploid State Plays a Tumor-Suppressive Role in the Liver. Dev Cell.

[5] Kreutz C, MacNelly S, Follo M, Waldin A, Binninger-Lacour P, Timmer J (2017). Hepatocyte Ploidy Is a Diversity Factor for Liver Homeostasis. Front Physiol.

[6] Fox DT, Duronio RJ (2013). Endoreplication and polyploidy: insights into development and disease. Development.

[7] Gentric G, Maillet V, Paradis V, Couton D, L’Hermitte A, Panasyuk G (2015). Oxidative stress promotes pathologic polyploidization in nonalcoholic fatty liver disease. J Clin Invest.

[8] Gentric G, Desdouets C (2015). Liver polyploidy: Dr Jekyll or Mr Hide?. Oncotarget.

[9] Margall-Ducos G, Celton-Morizur S, Couton D, Bregerie O, Desdouets C (2007). Liver tetraploidization is controlled by a new process of incomplete cytokinesis. J Cell Sci.

[10] Toyoda H, Bregerie O, Vallet A, Nalpas B, Pivert G, Brechot C (2005). Changes to hepatocyte ploidy and binuclearity profiles during human chronic viral hepatitis. Gut.

[11] Davoli T, de Lange T (2011). The causes and consequences of polyploidy in normal development and cancer. Annu Rev Cell Dev Biol.

[12] Donne R, Saroul-Ainama M, Cordier P, Celton-Morizur S, Desdouets C (2020). Polyploidy in liver development, homeostasis and disease. Nat Rev Gastroenterol Hepatol.

[13] Pandit SK, Westendorp B, de Bruin A (2013). Physiological significance of polyploidization in mammalian cells. Trends Cell Biol.

[14] Lin YH, Zhang S, Zhu M, Lu T, Chen K, Wen Z (2020). Mice With Increased Numbers of Polyploid Hepatocytes Maintain Regenerative Capacity But Develop Fewer Hepatocellular Carcinomas Following Chronic Liver Injury. Gastroenterology.

[15] Ghouri YA, Mian I, Rowe JH (2017). Review of hepatocellular carcinoma: Epidemiology, etiology, and carcinogenesis. J Carcinog.

[16] Golabi P, Fazel S, Otgonsuren M, Sayiner M, Locklear CT, Younossi ZM (2017). Mortality assessment of patients with hepatocellular carcinoma according to underlying disease and treatment modalities. Med (Baltim).

[17] Yang JD, Hainaut P, Gores GJ, Amadou A, Plymoth A, Roberts LR (2019). A global view of hepatocellular carcinoma: trends, risk, prevention and management. Nat Rev Gastroenterol Hepatol.

[18] Duncan AW (2013). Aneuploidy, polyploidy and ploidy reversal in the liver. Semin Cell Dev Biol.

[19] Goussev SA, Center SA, Randolph JF, Kathrani A, Butler BP, McDonough SP (2016). Clinical Characteristics of Hepatocellular Carcinoma in 19 cats from a Single Institution (1980–2013). J Am Anim Hosp Assoc.

[20] Lawrence HJ, Erb HN, Harvey HJ (1994). Nonlymphomatous hepatobiliary masses in cats: 41 cases (1972 to 1991). Vet Surg.

[21] Post G, Patnaik AK (1992). Nonhematopoietic hepatic neoplasms in cats: 21 cases (1983–1988). J Am Vet Med Assoc.

[22] Zhang S, Zhou K, Luo X, Li L, Tu HC, Sehgal A (2018). The Polyploid State Plays a Tumor-Suppressive Role in the Liver. Dev Cell.

[23] Wilkinson PD, Delgado ER, Alencastro F, Leek MP, Roy N, Weirich MP (2019). The Polyploid State Restricts Hepatocyte Proliferation and Liver Regeneration in Mice. Hepatology.

[24] Roberts EA, Letarte M, Squire J, Yang S (1994). Characterization of human hepatocyte lines derived from normal liver tissue. Hepatology.

[25] Anti M, Marra G, Rapaccini GL, Rumi C, Bussa S, Fadda G (1994). DNA ploidy pattern in human chronic liver diseases and hepatic nodular lesions. Flow cytometric analysis on echo-guided needle liver biopsy. Cancer.

[26] Fujimoto J, Okamoto E, Yamanaka N, Toyosaka A, Mitsunobu M (1991). Flow cytometric DNA analysis of hepatocellular carcinoma. Cancer.

[27] Lim S, Ganem NJ (2014). Tetraploidy and tumor development. Oncotarget.

[28] Ganem NJ, Storchova Z, Pellman D (2007). Tetraploidy, aneuploidy and cancer. Curr Opin Genet Dev.

[29] Pesavento PA, Jackson K, Hampson T, Munday JS, Barrs VR, Beatty JA. A Novel Hepadnavirus is Associated with Chronic Hepatitis and Hepatocellular Carcinoma in Cats. Viruses. 2019;11(10). 10.3390/v11100969 PMC6832243 31640283

[30] Gramantieri L, Melchiorri C, Chieco P, Gaiani S, Stecca B, Casali A (1996). Alteration of DNA ploidy and cell nuclearity in human hepatocellular carcinoma associated with HBV infection. J Hepatol.

[31] van Sprundel RG, van den Ingh TS, Guscetti F, Kershaw O, van Wolferen ME, Rothuizen J (2014). Classification of primary hepatic tumours in the cat. Vet J.

[32] Martins-Filho SN, Paiva C, Azevedo RS, Alves VAF (2017). Histological Grading of Hepatocellular Carcinoma-A Systematic Review of Literature. Front Med (Lausanne).

